# Effects of settled diatoms on autumn phytoplankton blooms in the Pacific Arctic: A process study of sediment resuspension in the Chukchi shelf

**DOI:** 10.1371/journal.pone.0357159

**Published:** 2026-08-28

**Authors:** Yuri Fukai, Amane Fujiwara, Kohei Matsuno

**Affiliations:** 1 Research Institute for Global Change, Japan Agency for Marine-Earth Science and Technology (JAMSTEC), Yokosuka, Kanagawa, Japan; 2 Field Science Center for Northern Biosphere, Hokkaido University, Hakodate, Hokkaido, Japan; 3 Faculty/Graduate School of Fisheries Sciences, Hokkaido University, Hakodate, Hokkaido, Japan; 4 Arctic Research Center, Hokkaido University, Sapporo, Hokkaido, Japan; NRSC: National Remote Sensing Centre, INDIA

## Abstract

Autumn phytoplankton blooms are increasingly observed in the Arctic Ocean due to more frequent strong wind events over open waters. A recent study on the Chukchi shelf suggested that diatoms in sediments can resuspend to the surface during such events, potentially acting as a seed population for blooms. To investigate how resuspended diatoms could influence the dynamics of autumn bloom, we conducted an *in situ* incubation experiment under two conditions: (1) only nutrients are supplied to and (2) nutrients and microalgae-containing bottom-sediments are inoculated to phytoplankton community. Both experimental conditions resulted in increased microalgal biomass, particularly diatoms. Sediment input enhanced initial biomass, accelerating the transition to the bloom state, whereas the photophysiological parameters, including the maximum specific growth rate and the maximum quantum yield of photosystem II, were similar in both treatments. When only nutrients were supplied, *Arcocellulus* spp. and common autumn diatoms in the Chukchi Sea, including *Rhizosolenia* spp. and *Leptocylindrus* spp., prevailed. Meanwhile, diatoms typically present on the seafloor, such as the *Chaetoceros socialis* complex, *Chaetoceros* spp., and *Thalassiosira* spp., dominated the assemblage when sediments were inoculated. These results indicate that sediment resuspension to the surface potentially influences autumn bloom characteristics, affecting bloom development rate, size structure, and diatom community diversity and composition.

## Introduction

In recent years, the Arctic Ocean has experienced environmental changes. An increase in open-water area and period associated with sea ice loss, together with enhanced phytoplankton concentrations led by the supply of new nutrients, drives an increase in net primary production [1,2]. Microalgae, including phytoplankton, ice algae, and benthic microalgae, are the major primary producers and are highly sensitive to shifts in ambient conditions. Variations in temperature, salinity, and CO_2_ concentrations, which are inconsistent in the Arctic due to rapid warming, interactively affect microalgal communities in terms of composition, biomass, and diversity [3,4]. Further, environmental changes directly affect microalgae and alter the phenology of primary producers. Changes in sea ice dynamics are key factors in controlling phytoplankton phenology, and earlier sea ice retreat results in changes in both phytoplankton bloom timing and magnitude [5,6]. Frequent autumn microalgal blooms [7,8] are another example of phenological changes in the Arctic related to sea ice dynamics. In other words, there are considerable primary productive events during autumn besides spring microalgal blooms. Delay in sea ice formation increases the open-water periods and the number of stormy days over open-waters. Such conditions lead to stronger wind-driven vertical mixing and upward supply of nutrients, thereby driving autumn phytoplankton blooms [7].

Notably, in the shelf region of the Pacific sector of the Arctic, the Chukchi shelf, autumn phytoplankton blooms have become more frequent over the past decade [8]. Mooring-based sediment traps capture high algal flux in the Chukchi Sea from August to October, indicating substantial autumn production [9]. Nishino et al. (2015) also reported that strong winds supplied nutrients to the surface, enhancing the primary production and biomass of large microalgae, such as diatoms, through ship-based observations at a fixed-station [10]. Further, a study using satellite observation suggested that autumn phytoplankton blooms may lead to a community predominated by larger phytoplankton than under conditions where no autumn bloom develops [11]. However, there is still a question about the autumn bloom development process: what is the initial population for autumn blooms?

Seafloor sediments in Pacific Arctic shelves contain abundant viable diatoms [12,13]. In particular, diatoms are highly accumulated in sediments in the Chukchi shelf, reflecting high seasonal primary production in the water column [13]. Diatoms in sediments are not dead yet; they, including resting spores, can germinate and resume growing by triggering light exposure [14]. They have high photophysiological plasticity to drastic light changes and can quickly resume their primary production even after more than nine months of darkness, as indicated by a laboratory experiment using sediment from the Chukchi shelf [15]. Thus, they can contribute to spring blooms in the coastal shallow Arctic Svalbard [16] and bottom-associated blooms in the Chukchi shelf [17] when enough light for the growth reaches the bottom. In addition, a recent study on the Chukchi shelf indicated the possibility that diatoms in sediments come up to the sunlit ocean surface during strong wind events. Some circumstantial evidence from mooring and ship-based observations suggests that the wind modulates the bottom current, which induces the resuspension of sediments and microalgae during autumn [18]. It was speculated that strong wind enhances the current within the benthic boundary layer, which entrains *Chaetoceros* resting spore-rich sediments and nutrients (including NH_4_) to the ocean interior, resulting in seeding potential for autumn microalgal blooms [18]. The concentration of diatoms in sediments in the Chukchi shelf is much higher than that in the water column during autumn [12], suggesting that once sediments are resuspended to the surface with sufficient light for germination and growth, they could act as sources for the initial population for autumn blooms. However, no study has investigated how the resuspension of sediments affects the following autumn bloom.

We conceptualized two possible scenarios of the initial process of autumn bloom: the no-sediment-resuspension scenario, in which only nutrients are supplied to the surface, and the sediment-resuspension scenario, in which both nutrients and sediments containing microalgae are transported from deeper layers. In this study, using *in situ* incubation experiments under two different conditions, we aimed to reveal the potential effects of sediment resuspension on the following autumn bloom characteristics.

## Materials and methods

**Fig 1:**
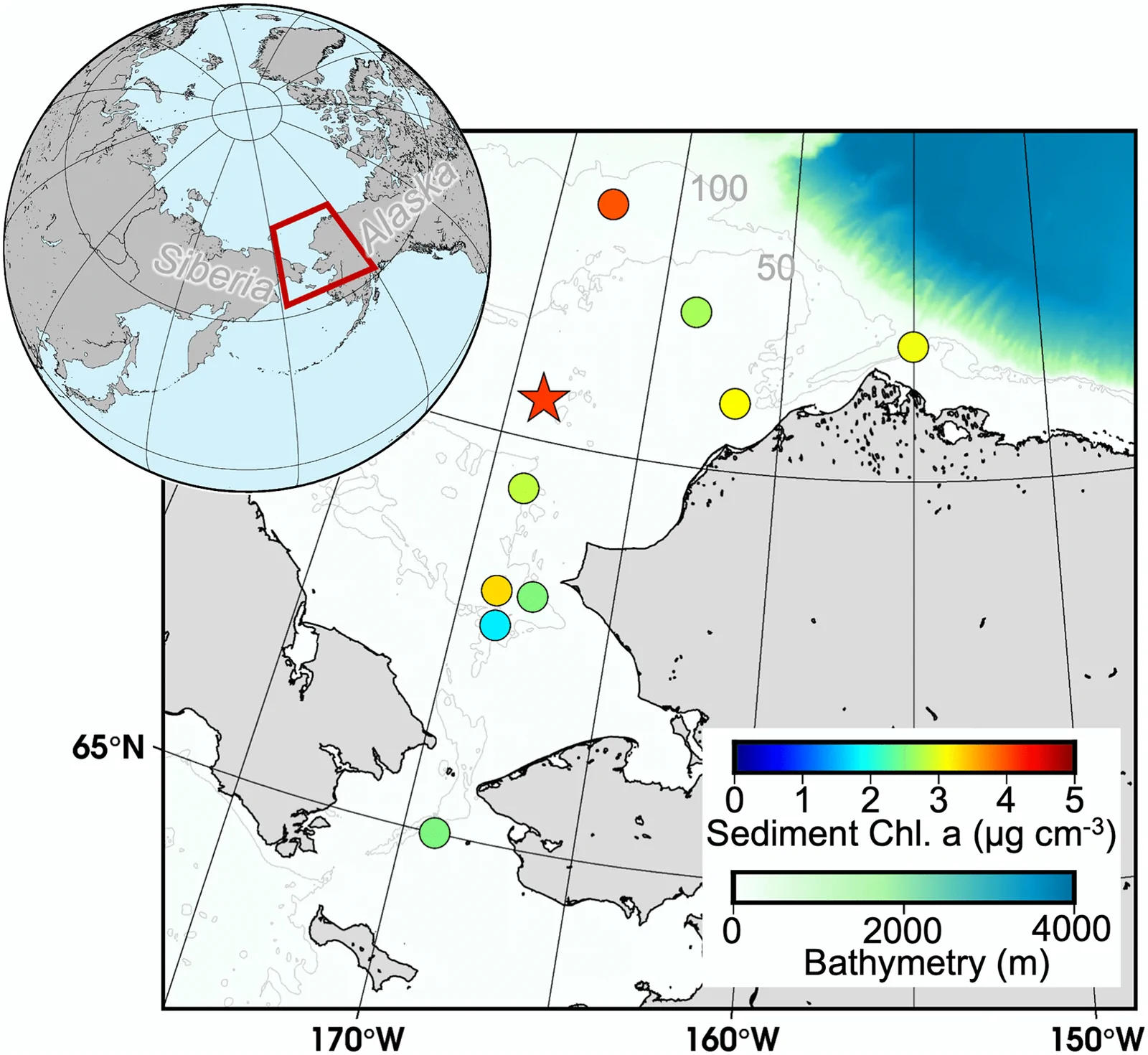
Horizontal distribution of Chl *a* concentration in surface sediments across the study area. Chl *a* concentration is expressed as µg Chl *a* per cm^3^ of the wet sediment. The star indicates the station from which seawater and sediment samples were collected for the *in situ* incubation experiments. Note that the circles and the star share the same color scale. This map was created using Generic Mapping Tools (GMT). Bathymetric data were obtained from the ETOPO1 Global Relief Model which is a public domain dataset provided by the National Oceanic and Atmospheric Administration (NOAA)(https://doi.org/10.7289/V5C8276M).

Samples of seawater and sediments for the experiment were collected during an Arctic cruise by the R/V *Mirai* (MR23-05C) on September 9, 2023, on the Chukchi shelf at 70.5°N, 168.75°W (bottom depth: 39 m) (Fig 1). Note that written permission for sample collection within the United States Exclusive Economic Zone (EEZ) was granted by the U.S. Department of State (Permit number: U2023-005). Seawater was obtained from the surface layer at 5 m, the subsurface chlorophyll *a* (Chl *a*) maximum (SCM) layer at 23 m, and the near-bottom layer at 32.9 m using Niskin-X samplers mounted on a CTD rosette system. The surface and SCM seawaters were subsampled through a 200-µm mesh to remove large zooplankton, and the near-bottom seawater was filtered using a filter capsule with a 0.2-µm pore size (ADVANTEC Disposable Capsule Filters, CCS-020-D1H). The sediment sample was retrieved using a bottom core sampler, ASYURA (Rigosha Co., Ltd., Japan), and the uppermost sediment layer was carefully subsampled using a spatula. Afterward, we added the sediment to the filtered near-bottom seawater to prepare the sediment-suspended seawater for subsequent experiments.

Using seawater and sediments, we set two types of incubation bottles to test the potential effect of sediment input on autumn microalgal blooms: (1) only nutrients are supplied to (the no-sediment-suspension condition) and (2) both nutrients and bottom-sediments containing microalgae are inoculated to (the sediment-suspension condition) phytoplankton community in the water column. For the bottles of condition (1), we combined 4-L surface seawater, 3-L SCM seawater, and 3-L of the sediment-suspended seawater filtered by a 0.2-µm filter capsule. Meanwhile, for condition (2), we added the unfiltered sediment-suspended seawater to a mixture of surface and SCM seawater to obtain a sediment concentration of 0.5 cm^3^ L^−1^. Each treatment was prepared in triplicate. Incubation was conducted for 8 days under *in situ* sea surface temperature and light conditions using an on-deck incubator. During the incubation, we monitored the concentration of nitrogen nutrients (NO_2_ + NO_3_ and NH_4_) and microalgal parameters, including the concentration of total and size-fractionated Chl *a* and phytoplankton pigments, diatom community composition, and the maximum quantum yield of photochemistry in photosystem II (i.e., *F*_v_/*F*_m_) (S1 Table).

Nutrients samples (~10 mL) were collected for on board measurement using colorimetric methods with a QuAAtro 2-HR system. For Chl *a* analysis, 100 mL and 200 mL seawater samples were collected to determine total and size-fractionated (>20, 2–20, and <2µm) concentrations, respectively, using a fluorometric method with a Turner Designs 10-AU fluorometer [19]. Nutrients and Chl *a* were measured as described in the cruise report [20]. For the analysis of microalgal pigments, 800-mL seawater was gently filtered through GF/F filters (ADVANTEC GF-75, pore size) under low vacuum pressure (< 0.013 MPa). The filtered samples were immediately flash-frozen in liquid nitrogen and stored at –80°C in an ultralow-temperature freezer until subsequent analysis. Pigment extraction was performed according to the method described by Suzuki et al. (2005) [21], and concentrations were quantified via high-performance liquid chromatography (HPLC) according to the protocols of Van Heukelem and Thomas (2001) and Fujiwara et al. (2014) [22,23]. Using a pigment-based chemotaxonomic tool with the R software package “phytoclass” version 1.0.0 [24], the Chl *a* biomass of 8 phytoplankton groups (diatoms, dinoflagellates, prymnesiophytes [haptophytes], chlorophytes, prasinophytes sensu lato, cryptophytes, and cyanobacteria) was estimated from 11 pigments (peridinin, 19’-butanoyloxyfucoxanthin, fucoxanthin, 19’-hexanoyloxyfucoxanthin, neoxanthin, prasinoxanthin, violaxanthin, alloxanthin, lutein, zeaxanthin, and Chl *b*) following a previous study in the Chukchi Sea [25]. As in the analytical settings of Fukai et al. (2025) [18], we set the iteration length to 500 and the step to 0.009 [24]. For the estimation, the minimum and maximum values for each pigment:Chl *a* ratio were calculated by multiplying the ratios from Zhuang et al. (2016) [25] by 0.1 and 3, respectively. Light microscopy and DNA metabarcoding techniques were used to analyze diatom communities. For microscopy, 100 mL of seawater was preserved using neutral buffered formalin to a final concentration of 2%, and 10–50 mL of fixed subsamples were concentrated 3- to 17-fold using an Utermöhl Chamber [26]. Using the concentrated samples, diatoms were enumerated and taxonomically identified at the species or genus level using an inverted light microscope, referring to Hasle and Syvertsen (1997) and Hoppenrath et al. (2009) [27,28], to determine the abundance-based composition. DNA was extracted using DNeasy PowerSoil Pro Kit (QIAGEN, Netherlands) from filters (PTFE membrane, 0.2-µm pore size) that had collected 700-mL seawater and were stored at –80°C. The V4 region of the 18S rRNA gene was sequenced using primers described by Piredda et al. (2017) [29]. Amplicon sequence variants (ASVs) were inferred from the obtained sequence data, and taxonomic annotation was performed using the Basic Local Alignment Search Tool against the Protist Ribosomal Reference database [30] (version 5.0.0) to identify diatom taxa and determine composition based on 18S rRNA gene abundance. Since the 18S rRNA gene copy number reflects diatom biovolume and biomass [31], the sequence data were used as a proxy for the relative biovolume and biomass of the diatom community. The protocols followed the methods described by Fukai et al. (2025) [18], and the obtained nucleotide sequence data have been deposited with links to BioProject accession number PRJDB40077 in the DDBJ BioProject database (https://www.ddbj.nig.ac.jp/bioproject/index-e.html). Diatom community diversity was assessed using ASVs by calculating the Shannon index (*H’*). The physiological states of microalgal communities were also assessed through the *F*_v_/*F*_m_ measured using a pulse-amplitude-modulated fluorometer (Water-PAM, Walz, Germany). To observe light conditions during *in situ* incubation, sea surface photosynthetically active radiation (PAR) was continuously monitored using a PAR sensor (PUV-510, Biospherical Instruments).

Horizontal sediment samples were collected using the ASYURA core sampler from 10 stations (Fig 1 and S1 Dataset) to measure Chl *a* concentration. The uppermost sediment layer was carefully subsampled using a spatula, and 1 cm^3^ of sediment was taken using a measuring scoop from the subsamples for subsequent analysis using a fluorometer (10-AU, TURNER DESIGNS), as described in the cruise report [20]. Chl *a* measurements were performed in triplicate for each subsample, and the average concentration was used for discussion.

To test statistical differences between the treatments in the initial concentrations of DIN and total Chl *a*, the maximum values of Chl *a* concentration, *F*_v_/*F*_m_, and Chl *a*-specific growth rate, and the earlier response of the Chl *a*-specific growth rate, Welch’s t-tests were performed. For the Shannon index (*H*’), statistical comparisons among treatments and sampling days were conducted using a one-way ANOVA followed by the Tukey-Kramer post hoc test.

## Results

Sediment Chl *a* concentration ranged from 1.80 to 4.10 µg per cm^3^ of wet sediment across the study area. The sediment used in the incubation experiment contained the highest Chl *a* concentration (Fig 1 and S1 Dataset). In terms of simple volume-based comparison, the sediment contained approximately three orders of magnitude more Chl *a* than the SCM seawater (1.02 µg L^−1^) used in the experiment.

**Fig 2:**
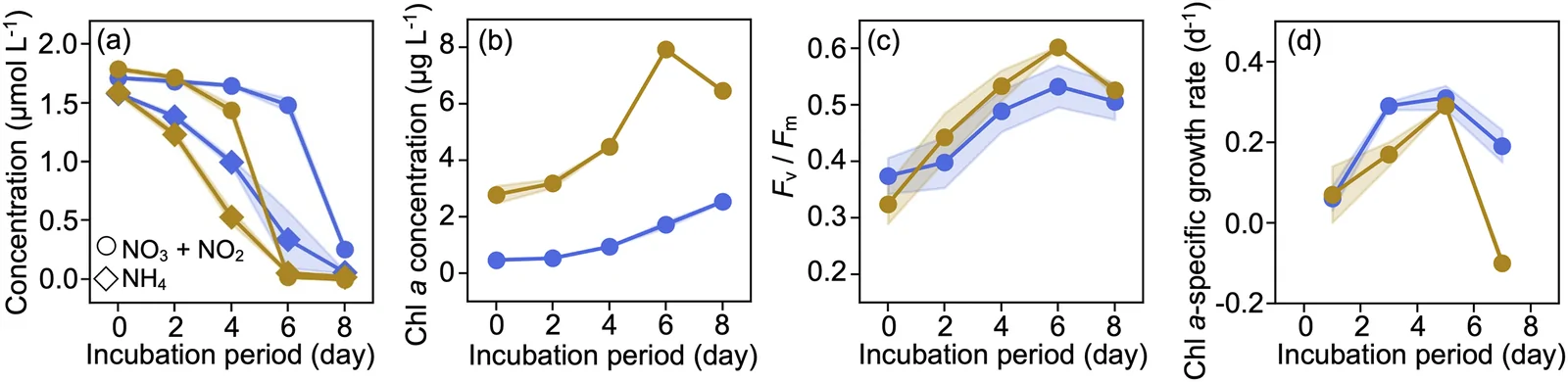
Temporal changes in concentrations of dissolved inorganic nitrogen, microalgal biomass, and physiological parameters during the incubation experiment. Time series of NO_3_ + NO_2_ and NH_4_ concentrations (a), Chl *a* concentration (b), maximum quantum yield of photosystem II (*F*_v_/*F*_m_) (c), and Chl *a*-specific growth rate (d). Blue and brown show the results for the no-sediment-suspension and sediment-suspension treatments, respectively; the shaded areas indicate the standard deviation from triplicate experiments.

**Fig 3:**
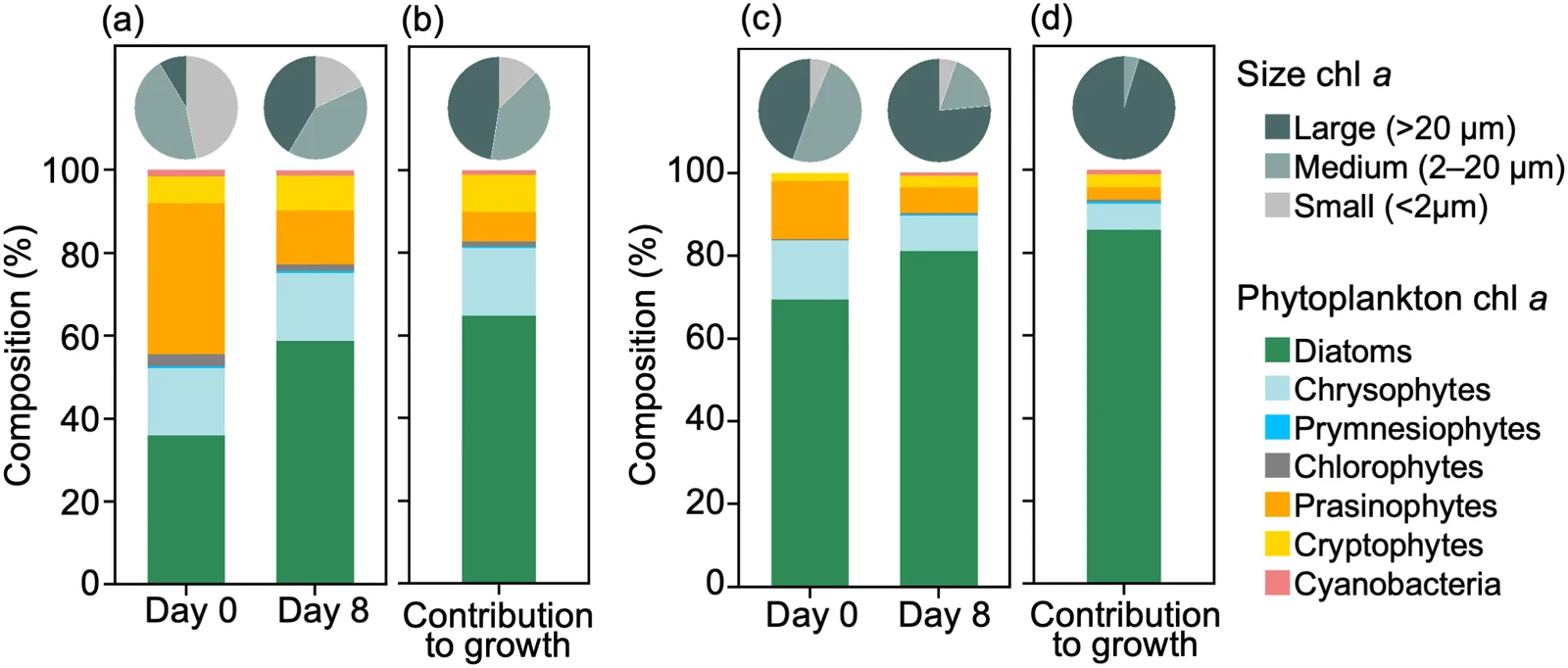
Composition of size-fractionated and phytoplankton Chl *a* and their contribution to biomass growth. The composition in the no-sediment-suspension treatment (a) and the sediment-suspension treatment (c) on incubation days 0 and 8. Contribution to growth indicates the proportion of phytoplankton contributing to the increase in Chl *a* biomass from day 0 to 8 in the no-sediment-suspension treatment (b) and the sediment-suspension treatment (d). The pie charts indicate the composition of size-fractionated Chl *a*, and the bar graphs indicate phytoplankton Chl *a* based on HPLC analysis.

During the incubation experiment, the daily maximum surface PAR ranged from 189 to 866 µmol photons m^−2^ s^−1^ (S2 Dataset), corresponding to a mean daily surface PAR of 7.16 mol photons m^−2^ d^−1^. The initial incubation conditions of both treatments were similar in terms of dissolved inorganic nitrogen (DIN) (t-test, *p* > 0.05) (Fig 2a, S2 Table and S3 Dataset). However, the input of sediments increased the initial microalgal biomass (t-test, *p* < 0.05) (Fig 2b, S2 Table and S3 Dataset), especially a large fraction of Chl *a*, which would be mostly from diatoms (Fig 3 and S4 Dataset). Microalgal biomass based on Chl *a* concentration increased during incubation in both treatments, except for the last two days of the sediment-suspended bottles (Fig 2b). For the last two days, DIN was depleted in the bottles with sediment whereas nitrate was still available (0.25 µmol L^−1^) in the treatment without sediment input (Fig 2a). Despite the different trends and maximum values (t-test, *p* < 0.05) (S2 Table) in the total Chl *a* concentration between both treatments, the time series of the physiological indicator—the maximum quantum yield of photochemistry in photosystem II (*F*_v_/*F*_m_)—showed similar trends, reaching the equivalent maximum values (0.53 and 0.60 in the no-sediment-suspension and sediment-suspension treatments, respectively) (t-test, *p* > 0.05) within a week (Fig 2c, S2 Table and S3 Dataset). In addition, specific growth rates based on the Chl *a* concentration ([µg L^-1^] [µg L^-1^]^-1^ d^-1^) for both treatments were comparable before nutrient depletion after day 6, although the rates observed from day 2–4 were higher in the no-sediment-suspension treatments (t-test, *p* < 0.05) (Fig 2d and S2 Table). The maximum specific growth rates based on the Chl *a* concentration in both treatments were similar from day 4–6 (t-test, *p* > 0.05), reaching 0.31 ± 0.03 and 0.29 ± 0.02 × 10^−1^ day^−1^, respectively (Fig 2d and S2 Table).

**Fig 4:**
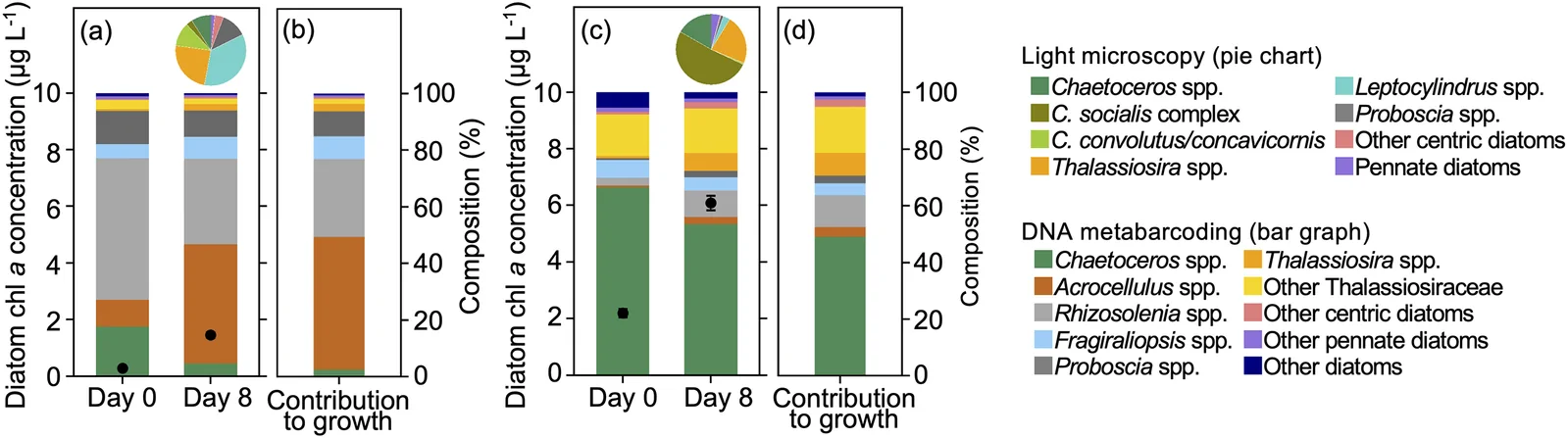
Diatom composition and contribution to biomass growth. Diatom composition in the no-sediment-suspension treatment (a) and the sediment-suspension treatment (c) on incubation days 0 and 8. Proportion of diatoms contributing to the increase in diatom Chl *a* biomass from day 0 to 8 in the no-sediment-suspension treatment (b) and the sediment-suspension treatment (d). Calculated using diatom Chl *a* concentrations determined by HPLC and community composition obtained from DNA metabarcoding. The pie charts and bar graphs indicate the diatom composition revealed by light microscopy and DNA metabarcoding, respectively.

**Fig 5:**
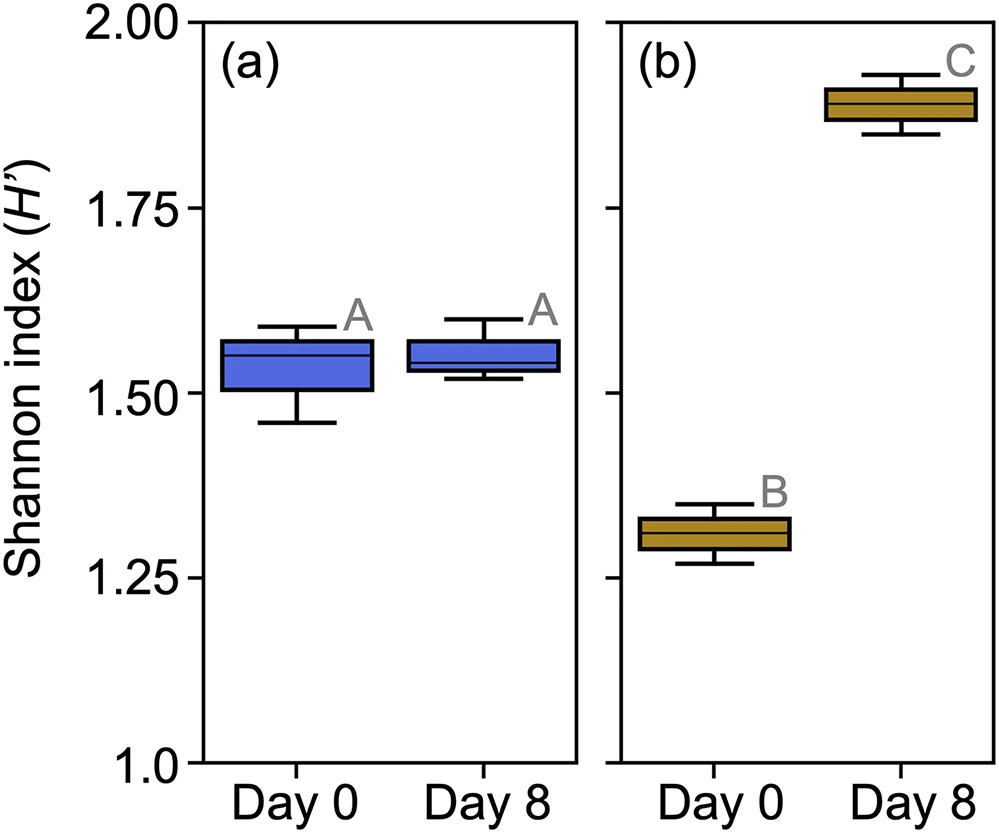
Diatom community diversity based on the Shannon index (*H’*) on days 0 and 8. Blue and brown box plots indicate the index in the no-sediment-suspension treatment (a) and the sediment-suspension treatment (b), respectively. The index was calculated using the ASV data of diatoms revealed by DNA metabarcoding. Different gray uppercase letters indicate statistically significant differences (*p* < 0.05) among treatments and sampling days (one-way ANOVA followed by the Tukey-Kramer test).

Size-fractionated Chl *a* and phytoplankton community structure compositions differed between the two treatments (Fig 3 and S3 Dataset). Especially, at the end of incubation, day 8, large-sized (>20 µm) phytoplankton and diatoms, respectively, reached up to 41% and 58.8% of Chl *a* biomass in the no-sediment-suspension treatment (Fig 3a) and 76% and 81.2% in the sediment-suspension treatment (Fig 3c). The differences between these treatment seemed to result from variations in the phytoplankton taxa responsible for growth. Large-sized phytoplankton and diatoms, respectively, contributed 47.5% and 64.7% to the growth of Chl *a* biomass in the no-sediment-suspension treatment (Fig 3b), whereas their contributions were 97.8% and 85.7% in the sediment-suspension treatment (Fig 3d). The diatom community structure differed between the two treatments at the beginning and end of the incubation (Fig 4). The genus *Rhizosolenia* accounted for nearly half (49.9%) of the initial diatom community in the no-sediment-suspension treatment, whereas their contribution was only 2.7% in the sediment-suspension treatment. On the other hand, the input of sediment enhanced the abundance of the genus *Chaetoceros*, accounting for 66.3% of the community. The genera *Leptocylindrus* and *Arcocellulus* actively grew in the no-sediment-suspension treatment, as confirmed by microscopy and DNA metabarcoding, respectively, representing 35.3% and 42.2% of the community at the end of the incubation (Fig 4a, S5 and S6 Datasets). In contrast, *Chaetoceros* spp., particularly *C. socialis* complex (as confirmed by microscopy), other Thalassiosiraceae, and *Thalassiosira* spp., contributed to the diatom community in the sediment-suspension treatment, account for more than half of the community (Fig 4c and S5 Dataset). As expected, the diatoms that contributed to the biomass increase differed between the treatments. *Arcocellulus* spp. and *Rhizosolenia* spp. were the major contributors in the no-sediment-suspension treatment (Fig 4b), whereas *Chaetoceros* spp. and *Thalassiosira* spp. dominated the contribution in the sediment-suspension treatment (Fig 4d). These contributions were estimated using diatom Chl *a* determined by HPLC together with community composition obtained from DNA metabarcoding as a proxy for relative biomass. Focusing on diatom community diversity, no clear trend in *H’* was observed in the no-sediment-suspension treatment (one-way ANOVA and Tukey-Kramer test, *p* > 0.05) (Fig 5a). Meanwhile, sediment input initially reduced *H’*, but diversity increased by the end of the incubation (one-way ANOVA and Tukey-Kramer test, *p* < 0.05) (Fig 5b).

## Discussion

The phenology of primary production in the Arctic is altering due to environmental changes attributable to sea ice reduction [6]. Prolonged open-water period leads to the Arctic Ocean where wind dominantly drives physical dynamics [32], resulting in favorable states for autumn phytoplankton blooms because of an increase in nutrient inputs in the upper layer [7]. Circumstantial evidence from mooring and ship-based observations in the Chukchi shelf revealed that diatoms in seafloor sediments can be transported near the surface [18]. As a process study, we further demonstrated the potential effects of resuspend sediments from the seafloor on determining characteristics and development of the autumn phytoplankton community. The initial diatom community in the no-sediment-suspension treatment was predominated by *Rhizosolenia* spp., one of the genera commonly observed in the Chukchi Shelf during autumn [33–35]. Meanwhile, because the Chukchi shelf sediments contain concentrated Chl *a* [36], comprising viable diatoms—such as *Chaetoceros socialis* complex, *Chaetoceros* spp., and *Thalassiosira* spp. [12,13,37]—whose communities are clearly distinct from those in the autumn water column [12,13,33], the input of sediment resulted in different initial communities. This study utilized both microscopy and DNA metabarcoding to evaluate diatom community composition. While microscopy remains a standard method yielding high-resolution taxonomic data (genus and species levels), the results of metabarcoding are strongly influenced by the choice of marker region and reference database [38]. Furthermore, the two methods reflect different biological metrics: microscopy-based cell counts are often dominated by small, chain-forming taxa (e.g., *Chaetoceros socialis* complex), whereas 18S rRNA gene copy numbers more closely reflect diatom biovolume and biomass [31]. Finally, different sample volumes can lead to analytical bias; the significantly larger volume processed for metabarcoding makes it more robust for assessing the overall community structure and detecting rare species [38]. These methodological differences were evident in our results, particularly regarding *Arcocellulus* spp., which can be attributed to its small cell size. The genus *Arcocellulus* spp. has been reported in the Arctic Ocean [39]. In this study, we showed its presence and high contribution to the diatom community in the no-sediment-suspension treatment using DNA metabarcoding; however, it was not detected via microscopy. Such a situation occurred in another study comparing the results from microscopy and DNA metabarcoding [40], meaning that they can be easily overlooked by microscopy due to their small cell size [27].

Sediment input led to a different composition and an increase in Chl *a* biomass in the initial community. The effect of sediment input extended into the subsequent period because diatoms in sediments keep their photophysiological capability and can quickly resume photosynthesis once they get sufficient light, even after long-term darkness [15]. Our incubation experiment was conducted in early September 2023, and the light intensity and day length were sufficient for diatoms from sediments to germinate and resume their photosynthesis. The mean surface daily PAR was 7.16 mol photons m^−2^ d^−1^, which was higher than the laboratory experiment condition (1.30 mol photons m^−2^ d^−1^) in Fukai et al. (2022) [15], confirming the high potential of diatoms in sediments on primary production. Further, Shiozaki et al. (2022) [17] showed that diatoms in sediments are associated with microalgal bloom near the bottom even under limited light availability (0.384 mol photons m^−2^ d^−1^). Therefore, even in the sediment-suspension treatment, microalgal biomass can increase and reach its maximum on day 6, which was earlier than in the no-sediment-suspension treatment. However, the photosynthesis potentials (*F*_v_/*F*_m_) and Chl *a*-specific growth rates of the two treatments were comparable during the incubation period, at least before the depletion of DIN. Therefore, we conclude that the faster transition to a bloom state in the sediment-suspension treatment was driven by the elevated initial biomass supplied by the sediment input, rather than by differences in the photophysiological state of the microalgal source communities. As expected, the elevated amount of Chl *a* depended on the amount of added sediment. To observe the subsequent response of the microalgal community, we added the sediment to achieve an additional Chl *a* concentration of approximately 2 µg L^−1^, a level frequently reached near the seafloor in this region [17]. Notably, this corresponds to assuming the resuspension of approximately 1.95 cm of sediment for a water depth of 39 m, which may exceed what typically occurs under realistic conditions.

Excessive addition of sediments reduced the diversity of the initial microalgal community in terms of *H’*. Sediment input would lead to an increased number of species, and reduced diversity would result from a decrease in species evenness. However, because this response depends on the amount of sediment added, the absolute values themselves would not be meaningful, and we need to further understand how the amount of suspended sediment affects the balance of increases in species richness and changes in evenness. Meanwhile, the 8-day incubation results suggest that the resuspension of sediments in the initial process of autumn blooms subsequently leads to a more diverse diatom community. Diatoms in the sediments can proliferate; thus, the evenness of species increased by the growth of various species together with the genus *Chaetoceros*, as indicated by their contributions to biomass growth, because species richness should remain largely unchanged. Such variations in the quality and characteristics of the diatom community, including its diversity, may play a pivotal role in influencing the efficiency of carbon pump transport [41] and zooplankton grazing activity [42].

This *in situ* experiment indicates a quick response by large-sized microalgae, diatoms, to an occasional nutrient input [10]. In other words, diatoms actively grew and contributed to the community growth, even in the no-sediment-suspension treatment, where Prasinophytes predominated in the initial microalgal community. However, the member of grown diatoms varied between the two treatments. Diatoms influenced by sediment input, such as *Chaetoceros socialis* complex, *Chaetoceros* spp., *Thalassiosira* spp., and other Thalassiosiraceae [13], contributed strongly to the diatom community growth in the sediment-suspension treatment. Meanwhile, common diatoms during autumn in the Chukchi Sea, such as *Rhizosolenia* spp. and *Leptocylindrus* spp. [33–35], and *Arcocellulus* spp., were the main contributors to growth in the no-sediment-suspension treatment. When evaluated in terms of Chl *a* biomass, *Rhizosolenia* spp. showed comparable concentrations in both treatments on day 8. *Arcocellulus* spp. did not exhibit a marked increase in the sediment-suspension treatment. Notably, that diatom community composition inferred from DNA metabarcoding is likely to reflect biovolume and biomass [31] rather than cell abundance, meaning that larger species are more readily detected as mentioned above. The elevated initial biomass of *Chaetoceros* spp. resulting from sediment input—a genus known for its high growth potential and ability to form substantial spring blooms in the Arctic [43,44]—may have proliferated preferentially over species originally present in the water column. Therefore, whether sediment resuspension occurs would determine the diatom community structure during the autumn blooms.

The Chukchi Sea, with a broad shallow shelf, shows one of the highest daily rates of primary productivity in the Arctic during spring and summer [45,46]. This results in a high level of particulate organic carbon flux across the global oceans [47], supporting patchy distributions of high benthic biomass, known as “biological hotspots” [48,49]. Such a strong relationship between the water column and seafloor is called “pelagic-benthic coupling,” which expresses the key biogeochemical features of the Chukchi shelf [48,49]. Moreover, settled diatoms in sediments can resuspend to the sunlit surface during strong wind events [18], which is an upward process from the seafloor to the water column within the “pelagic-benthic coupling” framework. Our study using the *in situ* incubation experiment during autumn further suggested that sediment resuspension to the euphotic layer have potential to alter the characteristics of subsequent primary production, including bloom development rate, size structure in the microalgal community, and diversity and genera/species composition in the diatom community. Because primary production in the Arctic ceases during autumn due to the extent of sea ice coverage and decreasing solar radiation, a shortened transition to the bloom state, driven by the elevated-baseline effect on initial biomass caused by sediment input, may have ecological benefits. In fact, the pulsed increase in diatom biomass benefits phytoplankton grazers [50,51], and food quality and food supply duration before winter diapausing are crucial for their developmental success [52]. Further investigation of their cascading impacts on ecosystems is necessary because the potential effects of autumn blooms driven by sediment resuspension on secondary producers may vary. Moreover, wind-induced increased primary production and sediment resuspension could lead to higher bacterial production and abundance in this region [53]. Therefore, functioning as integrated components, these sediments likely play a vital role in regional carbon cycling and marine ecosystems where water-column and seafloor interactions are highly dynamic.

In the shelf region of the Pacific Arctic, bathymetry, nutrient storage, and depth of nutricline [54], and diatom community in sediments [13] are regionally heterogeneous, and environmental factors related to fall blooms vary regionally. Meanwhile, the physical drivers of upper ocean dynamics shifted to a new normal with low sea ice after 2007, and wind mixing during a prolonged open water period gradually dominated upper ocean dynamics [32]. Given the geographical variability and ongoing environmental change, the process governing autumn phytoplankton blooms is highly complex. The dynamics of diatoms, one of the major primary producers of ecosystems, is a key component of this complex bloom process, and it should be interpreted within the pelagic–benthic coupling framework, a key feature of the Pacific Arctic shelf system.

## Conclusions

We demonstrated the potential effects of resuspended sediments on the autumn phytoplankton community and their developmental processes using *in situ* incubation experiments on the Chukchi shelf. The sediments contained concentrated viable diatom cells with high photosynthetic plasticity. Their addition increased initial biomass, accelerated the transition to bloom conditions, and altered the size structure and diatom assemblage composition. This sediment-driven process of autumn blooms may represent an important biogeochemical system in the shallow Pacific Arctic and should be further investigated in terms of its ecological significance and role in the carbon cycle.

## Supporting information

S1 Table Summary of parameters and sampling days. (PDF)

S2 Table Statistical comparison of parameters using Welch’s t-test. The table shows the comparison targets, sampling days, and resulting *p*-values.
(PDF)

S1 Dataset Sampling sites and chlorophyll *a* concentration in sediments. (XLSX)

S2 Dataset Sea surface photosynthetically active radiation during the incubation experiment. (XLSX)

S3 Dataset Concentrations of dissolved inorganic nitrogen, chlorophyll *a* concentration, and *F*_v_/*F*_m_. (XLSX)

S4 Dataset Chlorophyll *a* concentrations of phytoplankton groups estimated using HPLC analysis and a pigment-based chemotaxonomic approach. (XLSX)

S5 Dataset Diatom cell concentration estimated by microscopy. (XLSX)

S6 Dataset Sequence data and estimated composition of diatoms obtained via DNA metabarcoding. (XLSX)
