# Supplementary material for: Effects of settled diatoms on autumn phytoplankton blooms in the Pacific Arctic: A process study of sediment resuspension in the Chukchi shelf
Source: PLoS One. 2026 Aug 28;21(8):e0357159. doi: 10.1371/journal.pone.0357159 (PMC13524300; doi:10.1371/journal.pone.0357159)
Supplement: Supplementary file 1 [file pone.0357159.s001.pdf]

S1 Table. Summary of parameters and sampling days.

Parameters	Sampling day				
	Day 0	Day 2	Day 4	Day 6	Day 8
Nutrients	○	○	○	○	○
F_v / F_m	○	○	○	○	○
Total Chl *a*	○	○	○	○	○
Size Chl *a*	○				○
Pigments (HPLC)	○				○
DNA	○				○
Light microscopy					○
