# Supplementary material for: Effects of settled diatoms on autumn phytoplankton blooms in the Pacific Arctic: A process study of sediment resuspension in the Chukchi shelf
Source: PLoS One. 2026 Aug 28;21(8):e0357159. doi: 10.1371/journal.pone.0357159 (PMC13524300; doi:10.1371/journal.pone.0357159)
Supplement: Supplementary file 2 [file pone.0357159.s002.pdf]

S2 Table. Statistical comparison of parameters using Welch's t-test. The table shows the comparison targets, sampling days, and resulting *p*-values.

Parameters	Comparison target	Compared sampling day		*p*-value
		Treatment (1)	Treatment (2)	
DIN	Initial conc.	Day 0	Day 0	> 0.05
Total Chl *a*	Initial conc.	Day 0	Day 0	< 0.05
Total Chl *a*	Maximum conc.	Day 8	Day 6	< 0.05
F_v / F_m	Maximum value	Day 6	Day 6	> 0.05
Chl *a* specific growth rate	Earlier response	Day 2–4	Day 2–4	< 0.05
Chl *a* specific growth rate	Maximum rate	Day 4–6	Day 4–6	> 0.05
